# Trajectories of Social Participation and Its Predictors in Older Adults: Based on the CLHLS Cohorts from 2002 to 2018

**DOI:** 10.3390/ijerph20054260

**Published:** 2023-02-27

**Authors:** Chi Zhang, Yinan Zhao, Xi Chen, Xiaoyang Li, Qingcai Liu, Ruotong Peng, Yifei Chen, Hui Feng

**Affiliations:** 1 Xiangya School of Nursing, Central South University, Changsha 410013, China; 2 Xiangya-Oceanwide Health Management Research Institute, Central South University, Changsha 410013, China

**Keywords:** longitudinal, social participation, group-based trajectory model, older adults

## Abstract

Social participation is a key factor in achieving active aging. This study aimed to explore the trajectories and predictors of social participation changes among older adults in China. The data used in this study are from the ongoing national longitudinal study CLHLS. A total of 2492 older adults from the cohort study were included. Group-based trajectory models (GBTM) were used to identify potential heterogeneity in longitudinal changes over time and investigate associations between baseline predictors and trajectories for different cohort members using logistic regression. Four different trajectories of social participation were reported in older adults, namely, stable (8.9%), slow decline (15.7%), lower score with decline (42.2%), and higher score with decline (9.5%). On multivariate analyses, age, years of schooling, pension, mental health, cognitive function, instrumental activities of daily living, and initial social participation scores significantly impact the rate of change in social participation over time. Four trajectories of social participation were identified in the Chinese elderly population. Management of mental health, physical function, and cognitive function appear to be important in maintaining the long-term social participation of older people in the community. Early identification of factors influencing the rapid decline in social participation and timely interventions can maintain or improve social participation levels in older adults.

## 1. Introduction

The United Nations estimates that the number of people aged 60 and over will increase to about 2 billion worldwide by 2050 [1]. The number of older people is growing at an unprecedented rate and will accelerate in the coming decades, especially in developing countries. The aging of the population is gradually deepening, and the incidence of chronic diseases, cognitive dysfunction, functional disability, and mental disorders is on the rise. A study shows that 15.07 million Chinese aged 60 and above suffer from dementia, and 38.77 million suffer from mild cognitive impairment [2]. More than one in five people suffer from depression and anxiety, posing a huge challenge to our public health and healthcare system [3]. In 2002, the World Health Organization (WHO) proposed the concept of active aging and emphasized that the quality of life in seniors may be improved via the three channels of social participation, health, and safety, which help achieve the objective of healthy aging [4]. Among them, social participation is a key element, aimed at integrating older people into society.

According to Hsu and Levasseur et al., social participation refers to participation in social activities, such as paid or unpaid jobs, voluntary work, and social groups, which provide interaction with others in the community [5,6]. Social participation not only helps older adults maintain social connections after retirement and avoid social isolation, but it also improves physical and mental health by allowing them to reap the benefits of communication and interaction with others, as well as promoting their physical activity [7]. Zhou et al. [8] found that higher social participation can reduce the risk of dementia. In a study of 12,951 older Japanese, Kanamori et al. [9] showed that social participation can reduce the risk of functional disability. A 4-year longitudinal study by Du et al. [10] showed that a lack of formal and informal social participation increased the risk of depressive symptoms in Chinese older adults. According to the study of Zhao et al. [11], a decrease in social participation is associated with an increase of loneliness. Social participation has a profound impact on the lives of the elderly and plays an important role in their physical and mental health and social integration.

Some studies have explored the influencing factors on social participation of the elderly from individual, environmental and social aspects. Chen et al. [7] showed that social demographic characteristics such as gender, age, educational level, marital status, and living pattern could affect the social participation of the elderly. Owari et al. [12] showed that psychological distress may affect the social participation of older adults one year later. A systematic review shows that the main barriers to and facilitators of social participation of older adults are demographic, individual/internal, environmental/infrastructure, and social network factors [13]. However, there is a lack of research on the influencing factors on the key points in the trend of social participation. Many researchers have explored the concept and definition of social participation, either as an independent variable to explore the impact of social participation on outcome indicators such as physical function, cognitive function, and mental health of the elderly, or as a mediating factor to explore its role in the path of various health outcomes. However, the longitudinal effects of older adults’ existing health problems on social participation are lacking (e.g., cognitive function, mental health). Therefore, this study will longitudinally explore the influencing factors on the change trajectory of social participation of the elderly from multiple aspects.

The trajectory analysis of social participation in older adults can provide important information for determining whether the trajectory occurs and its key points, which is of great benefit for maintaining the health of the elderly. At present, there are many cross-sectional studies on the level of social participation of the elderly and its influencing factors, but there is a lack of studies on the change trajectory of social participation using machine learning methods. Therefore, this study aimed to explore the trajectory changes and critical points of social participation, and to hypothesize that demographic characteristics, physical and mental health status are correlated with changes in the trajectory of social participation of the elderly in China.

## 2. Materials and Methods

### 2.1. Study Design and Participants

Data were obtained from the 2002, 2005, 2008, 2011, 2014, and 2018 waves of the Chinese Longitudinal Healthy Longevity Survey (CLHLS). In the present study, only participants aged 60 years or older were included at baseline, and we excluded participants who were missing more than 2 waves in the subsequent 16 years. The CLHLS is one of the largest national longitudinal studies for investigating the health of older Chinese adults. A total of 23 Chinese provinces (Beijing, Tianjin, Hebei, Shanxi, Liaoning, Jilin, Heilongjiang, Shanghai, Jiangsu, Zhejiang, Anhui, Fujian, Jiangxi, Shandong, Henan, Hubei, Hunan, Guangdong, Guangxi, Chongqing, Sichuan, Shanxi, and Hainan) were randomly included, and the sampling frame covered about 85% of the total population of China, and about half of the cities/counties in each province were selected as primary survey units. Since 1998, eight follow-up visits were conducted every 2 to 3 years. To reduce attrition, new participants are continually enrolled as death and lost-to-follow-up are inevitable. Trained interviewers collected data through face-to-face surveys with a structured questionnaire. Proxy respondents, usually a spouse or other close family member, were interviewed when the participants were unable to answer questions, but questions regarding cognitive function and mood were answered by participants themselves. The CLHLS also adopted a targeted random-sample design to ensure representativeness, through interviewing approximately equal numbers of male and female nonagenarians, octogenarians and young-old living ”near” the centenarians (“near” means in the same village or street, if available, or in the same sampled county or city) [14]. The CLHLS survey covers mental health, cognitive function, social participation, diet and nutrition, lifestyle habits, socioeconomic status, family structure, intergenerational relationships, etc. Details of the study design and high data quality have been reported elsewhere [15].

### 2.2. Measurements

#### 2.2.1. Dependent Variables

In the construction of the index system of social participation, 10 activities in the CLHLS questionnaire are selected. According to the research of Wei [16] and Wang [17], social participation can be divided into cognitive activities, physical activities, and social activities. Selecting “read newspapers/books”, “play cards/mah-jong” and “watching TV and listen to radio”, three questions represent cognitive activities. Selecting “housework”, “personal outdoor activities”, “garden work”, “raise domestic animals/pets” and “exercise or not at present?”, five questions represent physical activities. Selecting “Do you take part in some social activities” and “traveling beyond home city/county in the past two years”, two questions represent social activities. In addition, since not many elderly people travel, this question is simplified to “Have you traveled in the last two years?”. Therefore, the total score of the questionnaire is 0-34. The higher the score, the better the social participation.

#### 2.2.2. Independent Variables

##### Health-Related Factors

Health-related factors include body impairment (hearing and visual function), self-reported quality of life (high, low), self-reported health (healthy, unhealthy), cognitive function, disability status, and mental health. Cognitive function was measured by the Chinese version of the modified Mini-Mental State Examination (CMMSE). The scale contains 12 questions in 5 dimensions: orientation, registration, attention and calculation, recall and language. The score ranged from 0 to 23 points, and higher scores indicated better cognitive function. The internal consistency of CMMSE has been verified in previous study [18]. Disability status was measured by instrumental activities of daily living (IADL) and activities of daily living (ADL). IADL was made up of eight items (visiting neighbors, shopping, making food, washing clothes, walking 1 km, carrying 5 kg weight, crouching and standing three times, and taking public transport). ADL was rated using six items (bathing, dressing, toileting, indoor transferring, continence, and feeding). Indoor transferring refers to whether an elderly person is able to get in and out of bed, and sit on or stand up from a chair or stool. Each item has three possible with three answers, i.e., “no limitation (0)”, “a little difficulty (1)”, and “unable to do (2)” [19]. Mental health was measured using seven items, each with five response levels. We reverse-coded the negatively oriented questions, including “feel fearful or anxious”, “feel lonely and isolated”, “feel useless with age”. The score ranges from 0 to 28 points, with a higher score suggesting a greater degree of mental health.

##### Lifestyle Factors

Lifestyle factors include smoking (yes, no), drinking (yes, no), fruit and vegetables intake. The frequency of fruit and vegetables intake was recorded as “almost every day (3)”, “except winter (2)”, “occasionally (1)” or “rarely or never (0)”.

##### Socio-Demographic Variables

According to previous studies, we selected some socio-demographic characteristics as the covariates, including age, gender (male, female), ethnic group (Han Chinese, minorities), current marital status (currently married, separated, divorced, widowed, and never married), years of schooling, residence (city, town, rural), co-residence of interviewee (with household member(s), alone, in an institution), pension (yes, no), financial sources (yes, no). Details of all variables are in Table 1.

### 2.3. Statistical Analysis

Descriptive statistical analysis was used to describe the basic characteristics of the participants. 

A Group-based trajectory model (GBTM) was adopted to explore the heterogeneity of social participation trajectories of the elderly. GBTM, as a mixed model, identifies clusters of individuals with similar trajectories through maximum likelihood estimation, and identifies several subgroups with different trajectory types from the population; the best-fit model was based on a Bayesian information criterion and the presence of at least 5% of participants for each trajectory to ensure stable estimation of each trajectory [20,21]. 

Multinomial logistic regression was used to analyze the influence of individual characteristics on the trajectory of social participation of the elderly. For all outcomes, the differences in trajectory groups from the final model among different participants’ characteristics were assessed using univariate analysis. Variables with *p* < 0.05 in univariate analysis were included in the multinomial logistic regression to determine independent predictors of each trajectory group.

Stata SE 17.0 and R studio were used for all statistical analysis. Two-sided *p* < 0.05 was considered statistically significant for all analysis.

## 3. Results

### 3.1. Participant Characteristics

The baseline characteristics of the participants for different trajectory groups are shown in Table 2. We included community-dwelling older adults aged 60 years or older at baseline (n = 16,064) and excluded participants who were missing more than two waves in the subsequent 16 years of follow-up data (n = 13,572). Finally, a total of 2492 older adults from the cohort study were included. The included older adults were younger, predominantly female, and higher educated than the excluded (all *p* < 0.05). See Table S1 for details. 

### 3.2. The Trajectories of Social Participation

**Figure 1:**
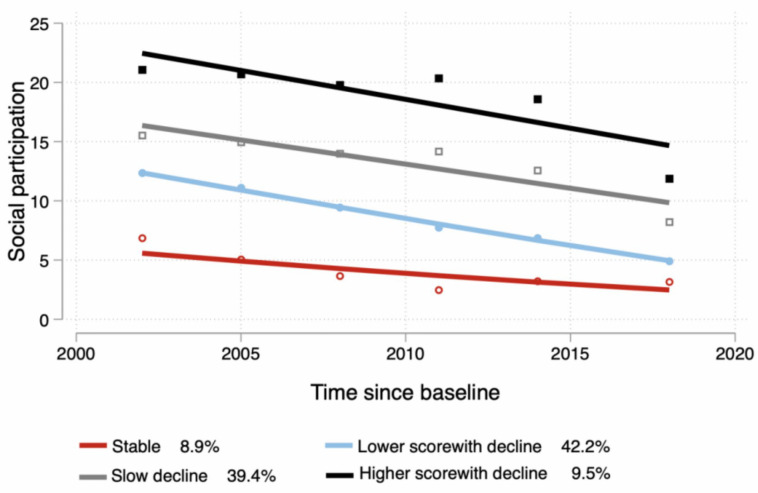
Trajectories in social participation.

From the trajectory model of the data at seven time points, four to seven levels were identified, depending on the parameter settings. By comparing the Akaike information criterion (AIC) and Bayesian Information Criterions (BIC) coefficient, we selected the four trajectories’ model, as is shown in Figure 1. The first group had lower social participation scores and was more stable during the 16-year follow-up period, which we defined as “stable” (n = 206, 8.9%). The second group had a higher baseline social participation score, but then showed a “slow decline” (n = 994, 39.4%). Compared to the first two groups, the third and fourth groups showed a rapid decline, but the third group had higher scores for baseline social participation, labeled “higher score with decline” (n = 211, 9.5%), while the fourth group had lower scores, labeled “lower score with decline” (n = 1081, 42.2%).

### 3.3. Predictors of Social Participation Trajectories

In Table 3, we report the relationships between the time and 16-year changes in social participation changes (slopes) for the covariates. Compared to the stable group, the older the age, both in the middle-aged (Slow decline: OR 0.20, 95%CI 0.10–0.42; Higher score with decline: OR 0.20, 95%CI 0.42–0.88) and older age groups (Lower score with decline: OR 0.23, 95% CI 0.11–0.48; Slow decline: OR 0.04, 95% CI 0.02–0.09; Higher score with decline: OR 0.01, 95% CI 0.02–0.09), the faster the rate of decline in social participation. Meanwhile, higher education level, sufficient pension, better physical function and cognitive function and initial social participation are the predictors of rapid decline in social participation during the following 16 years.

### 3.4. Sensitivity Analyses

In the Supplementary Materials Figure S1, we report results limited to participants who completed all follow-up visits, i.e., respondents who were present at all six measurement points (n = 704). In general, completers reported higher initial levels of social participation and slower rates of decline; there were fewer predictors associated with initial levels of social participation and rates of change; nevertheless, the overall findings remained unchanged.

## 4. Discussion

Based on nationally representative data on Chinese elderly, in this study, four potential categories were identified by using GBTM: stable, slow decline, lower score with decline, and higher score with decline. The model indicated that, over the 16 years of follow-up, there was a general decreasing trend in the average level of social participation of older adults. Very few respondents showed the lowest level of social participation in the baseline and maintained their level of social participation relatively well. Over half of older adults have a rapid decline in social participation. In addition, the results of the multivariate analysis showed that higher years of schooling, better mental health, higher levels of social participation at baseline, greater physical function, and better cognitive function may slow the decline. Conversely, we also found that higher age and no pension, may show a more rapid decline in social participation.

Social participation is a highly valued concept among older people as it is one of the most important components of older people’s health [6] and a key component of many conceptual models of older people’s functioning [22]. Several studies have shown that illness, mortality, and quality of life in older people are related to their social participation [6]. The results of this study are consistent with previous studies in South Korea, where the average trajectory of older people’s social participation showed a decline, mostly moderate or rapid [23]. However, in contrast to the results of another study in China [24], which analyzed 8117 participants, it was found that one group showed an upward trend over the 16-year follow-up period. The different results may be owing to the variable social engagement factors in this study, which was assessed by five dichotomous indicators: marital status; living arrangements; availability of help when needed, availability of confidants, and participation in social activities. In the social activities item, Ye’s study used a dichotomous variable [24], but our study used an overlay of the number of all activities. The number of activities participated in is an influencing factor in social participation, with a greater variety of participants representing a higher level of social participation among older people [25].

The results of this study show that age is strongly associated with a decline in the trajectory of social participation. The results of Buffel et al.’s [26] analysis of data from Belgian aging studies are highly consistent with this study, in that increasing age can significantly decrease/lower social participation. Luo et al. [27] also showed that advanced age was significantly associated with low social participation in a survey of 3456 older people aged 60 and over in western China. The role of pensions in social participation has rarely been explored in existing studies. This study points to No pension as another risk factor for a rapid decline in social participation, which is consistent with Lin’s [28] findings that older people with pensions are more involved in volunteer work. Older people with pensions tend to be at a higher economic level than those without pensions. He et al. [29] surveyed 2644 rural older people aged 60 and over and showed that those with high personal income were more willing to undertake social participation activities. Pensions, as the main source of income for older people, can improve the income structure of the elderly group and enhance their health. However, most rural residents have a weak awareness of insurance coverage and consider it non-essential. Some studies have shown that rural older people with pensions will have less paid work and more leisure time to spend with family or friends [30], which will increase the frequency of social participation. Therefore, social security departments should increase their efforts to promote the social pension insurance system in rural areas to increase the participation rate of rural residents and ensure a healthy later life.

This study also showed that low education level was a contributing factor to the declining rate of social participation. The findings of He et al. [29] were consistent with this study: older people with higher education levels tended to participate in more social participation activities. Lin’s [28] study based on the China Health and Retirement Longitudinal Study also showed that, the higher the education level of older people, the higher the frequency of their social participation. Although the educational level of older people is unchangeable, we can encourage them to participate in community activities for the elderly, universities for the elderly and other forms of education. Communities should also consider the actual needs of older people, provide friendly activity centers and infrastructure, and actively engage in senior education activities to increase skills, make friends, and promote social participation. 

In addition, this study showed that good mental health, IADL, and cognitive function were all protective factors in delaying the decline in social participation. A qualitative study of frail older adults [31] showed that declining functioning was a key barrier to social participation, such as slow reactions and poor mobility. A systematic evaluation also indicated that older adults with health problems such as depression or psychological dependence and physical and cognitive dysfunction were more likely to reduce their social participation activities, which is consistent with the findings of this study [32]. Xin et al. [33] also indicated that positive psychological characteristics may motivate older adults to be active in social participation activities. Furthermore, this study also found that baseline social participation levels were the most important protective factor for declining social participation among older adults. Social participation promotes older people’s psychological well-being, improves cognitive and physical functioning, and increases well-being [7,9,34], and these positive factors may in turn contribute to the long-term maintenance of social participation. Recent evidence recommends [35] that older people should be actively involved in cognitive activities involving memory, attention, calculation, and judgement (e.g., playing mahjong, group games, volunteering, etc.) and leisure activities (e.g., pet ownership, gardening, etc.), which may reduce the risk of developing cognitive impairment. Therefore, social organizations should emphasize the importance of social participation to older people and encourage them to take part in social activities regularly.

It is worth noting that previous studies have found that older people living in rural areas have a lower frequency of social participation compared to urban areas [29,36,37]. However, this study found no statistically significant change in the trajectory of older people’s place of residence and social participation. The reason for this may be nationwide efforts to build new rural areas, raise awareness of the importance of social participation among older people in rural areas, and build infrastructure extensively, etc., thus promoting regular participation in group social participation activities such as square dancing and tai chi. Moreover, this study also found that self-reported health status was not associated with statistically significant changes in the trajectory of social participation. However, Lin’s [28] findings suggest that older people with good self-reported health are more likely to participate in recreational activities. A mixed study of rural older people also showed [38] that good self-reported health was significantly and positively associated with social participation, which is inconsistent with the findings of the present study. This may be because social participation in this study included cognitive activities such as reading books and newspapers, watching television, and listening to the radio, etc. These cognitive activities can be performed at home without the need for activities or going out, even for older people with poor self-reported health status.

### Strengths and Limitations

The strengths of this study include, first, that CLHLS is a national survey and covers 85% of the total population in China, signifying excellent representativeness. The results can be generalized. Second, we used GBTM to analyze the trajectory changes of the social participation of the elderly, which can deal with the overall heterogeneity of the development trajectory. Third, we adjusted covariates, such as age and years of schooling, from a longitudinal perspective to explore their role in different trajectories of social participation.

However, this study has several limitations. First, the lack of a universal and available scale of social participation in the CLHLS may limit our findings. Second, this study did not consider the political participation of the elderly, which can enrich the connotation of social participation of the elderly in future studies. Third, the variables used in the study were measured by self-report, which may lead to information bias. Despite these limitations, we believe that the results can be used to guide the development of social participation intervention strategies for older adults. 

## 5. Conclusions

This study identified four trajectories of social participation among adults aged 65 years and older in China. Over the 16 years of follow-up, there was a general decreasing trend in the average level of social participation of older adults. Our study shows that years of schooling, mental health, level of social participation at baseline, physical function, and cognitive function are protective factors for social participation in older adults. Age and no pension are barriers to social participation. Early identification of factors influencing the rapid decline in social participation, understanding the heterogeneity of social participation trajectories, and timely adoption of targeted interventions can maintain or improve social participation levels in older adults, improve the quality of life, and promote healthy aging. 

## Figures and Tables

**Table 1:** Variables and their measurement.

Variables	Measurement
**Dependent variables**	
Social participation	0–34
**Independent variables**	
Hearing	0–3
Visual function	0–3
Self-reported quality of life	1 = high, 2 = low
Self-reported health	1 = healthy, 2 = unhealthy
CMMSE	0–23
ADL	0–12
IADL	0–16
Mental health	0–28
Currently smoking	1 = yes, 2 = no
Currently drinking	1 = yes, 2 = no
Fruit and vegetables intake	0–6
**Socio-demographic variables**	
Age	Continuous variable
Gender	1 = male, 2 = female
Ethnic group ^1^	1 = Han Chinese, 2 = minorities
Current marital status	1 = currently married, 2 = separated, 3 = divorced, 4 = widowed, 5 = never married
Years of schooling	Continuous variable
Residence	1 = city, 2 = town, 3 = rural
Co-residence of interviewee	1 = with household member(s), 2 = alone, 3 = in an institution
Pension	1 = yes, 2 = no
Financial sources	1 = yes, 2 = no

^1^: The Chinese nation is a big family made up of 56 ethnic groups, most of whom belong to the Han Chinese, while a small number belong to minorities. ADL: Activities of daily living; IADL: Instrumental activities of daily living; CMMSE: Chinese version of modified Mini-Mental State Examination.

**Table 2:** Baseline characteristics of the total sample and the sample by different trajectory groups.

Variables	Total (n = 2492)	Trajectory Group			
		Stable (n = 206)	Lower Score with Decline (n = 1081)	Slow Decline (n = 994)	Higher Score with Decline (n = 211)
**Socio-demographic variables**					
Age, Mean (SD)	74.69 (7.81)	83.64 (8.21)	76.42 (7.59)	71.74 (6.21)	71.04 (5.44)
Gender					
male	1171 (47.1)	65 (31.6)	426 (39.4)	526 (52.9)	157 (74.4)
female	1318 (52.9)	141 (68.4)	655 (60.6)	468 (47.1)	54 (25.6)
Ethnic group ^1^					
Han Chinese	2304 (92.5)	184 (89.3)	985 (91.1)	934 (94.0)	201 (95.3)
Minorities	188 (7.5)	22 (10.7)	96 (8.9)	60 (6.0)	10 (4.7)
Current marital status					
currently married	1352 (54.3)	54 (26.2)	484 (44.8)	656 (66.0)	158 (74.9)
separated	69 (2.8)	4 (1.9)	36 (3.3)	23 (2.3)	6 (2.8)
divorced	19 (0.8)	/	8 (0.7)	10 (1.0)	1 (0.5)
widowed	1033 (41.5)	146 (70.9)	539 (49.9)	302 (30.4)	46 (21.8)
never married	19 (0.8)	2 (1.0)	14 (1.3)	3 (0.3)	/
Years of schooling, Mean (SD)	2.71 (5.89)	1.11 (7.03)	1.63 (5.58)	3.32 (5.72)	6.96 (4.50)
Residence					
city	419 (16.8)	30 (14.6)	110 (10.2)	178 (17.9)	101 (47.9)
town	532 (21.3)	45 (21.8)	241 (22.3)	198 (19.9)	48 (22.7)
rural	1541 (61.8)	131 (63.6)	730 (67.5)	618 (62.2)	62 (29.4)
Co-residence of interviewee					
with household member(s)	2146 (86.1)	168 (81.6)	873 (80.8)	904 (90.9)	201 (95.3)
alone	305 (12.2)	33 (16.0)	177 (16.4)	87 (8.8)	8 (3.8)
in an institution	41 (1.6)	5 (2.4)	31 (2.9)	3 (0.3)	2 (0.9)
Pension					
yes	543 (21.8)	10 (4.9)	119 (11.1)	265 (70.6)	149 (70.6)
no	1947 (78.1)	196 (95.1)	960 (88.9)	729 (73.3)	62 (29.4)
Financial sources					
yes	2001 (80.3)	140 (68.0)	841 (77.8)	825 (83.1)	195 (92.4)
no	490 (19.7)	66 (32.0)	240 (22.2)	169 (16.9)	16 (7.6)
**Independent variables**					
Self-reported quality of life					
high	2315 (92.9)	169 (82.0)	988 (91.4)	952 (95.8)	206 (97.6)
low	152 (6.1)	22 (10.7)	84 (7.8)	41 (4.1)	5 (2.4)
Self-reported health					
healthy	2209 (88.6)	156 (75.7)	936 (86.6)	914 (92.0)	203 (96.2)
unhealthy	259 (10.4)	35 (17.0)	137 (12.7())	79 (7.9)	8 (3.8)
Currently smoking					
yes	626 (25.1)	34 (16.5)	248 (22.9)	275 (27.7)	69 (32.7)
no	1866 (74.9)	172 (83.5)	833 (77.1)	719 (72.3)	142 (67.3)
Currently drinking					
yes	595 (23.9)	33 (16.0)	228 (21.1)	270 (27.2)	64 (30.3)
no	1896 (76.1)	173 (84.0)	853 (78.9)	724 (72.8)	147 (69.7)
Hearing, Mean (SD)	2.88 (0.47)	2.49 (0.96)	2.88 (0.46)	2.96 (0.27)	2.96 (0.24)
Visual function, Mean (SD)	2.83 (0.49)	2.43 (0.91)	2.82 (0.49)	2.91 (0.34)	2.95 (0.25)
Fruit and vegetables intake, Mean (SD)	3.70 (1.21)	3.22 (1.28)	3.56 (1.13)	3.79 (1.21)	4.45 (1.24)
Mental health, Mean (SD)	19.89 (3.91)	18.5 (3.78)	19.16 (3.84)	20.47 (2.73)	22.30 (3.55)
ADL, Mean (SD)	11.89 (0.64)	11.41 (1.62)	11.90 (0.53)	11.95 (0.32)	11.96 (0.27)
IADL, Mean (SD)	14.58 (2.95)	10.28 (5.28)	14.43 (2.77)	15.40 (1.64)	15.68 (1.11)
CMMSE, Mean (SD)	20.60 (2.48)	16.70 (6.44)	20.31 (3.35)	21.40 (2.18)	22.14 (1.42)
**Dependent variables**					
Baseline social participation, Mean (SD)	13.95 (5.58)	6.37 (4.72)	12.26 (4.38)	15.73 (4.33)	21.60 (3.93)

^1^: The Chinese nation is a big family made up of 56 ethnic groups, most of whom belong to the Han Chinese, while a small number belong to minorities. ADL: Activities of daily living; IADL: Instrumental activities of daily living; CMMSE: Chinese version of modified Mini-Mental State Examination.

**Table 3:** Risk factors of trajectories (relative risk ratios and 95% confidence interval).

Baseline Variable	Group 2		Group 3		Group 4	
	Lower Score with Decline		Slow Decline		Higher Score with Decline	
**Socio-demographic variables**						
Age (Ref. 60–70)						
71–80	0.52	(0.25–1.05)	0.20 ***	(0.10–0.42)	0.20 ***	(0.42–0.88)
Above 80 above	0.23 ***	(0.11–0.48)	0.04 ***	(0.02–0.09)	0.01 ***	(0.02–0.09)
Female	1.29	(0.80–2.08)	1.12	(0.68–1.87)	0.66	(0.34–1.28)
Han Chinese ^1^	0.68	(0.35–1.30)	0.51	(0.24–1.06)	0.35	(0.12–1.06)
Single	0.72	(0.44–1.17)	0.62	(0.37–1.04)	0.78	(0.39–1.55)
Years of schooling	1.12	(0.99–1.26)	1.16 *	(1.03–1.30)	1.19 **	(1.11–1.34)
Residence (Ref. city)						
Town	1.38	(0.70–2.73)	0.87	(0.41–1.84)	0.49	(0.21–1.19)
Rural	1.65	(0.90–3.03)	1.24	(0.63–2.43)	0.59	(0.26–1.36)
Co-residence of interviewee (Ref. with household member (s))						
Alone	1.12	(0.64–1.93)	0.76	(0.41–1.42)	0.48	(0.16–1.40)
In an institution	1.76	(0.90–3.03)	0.21	(0.04–1.19)	0.76	(0.08–7.13)
No pension	0.92	(0.58–1.47)	0.60	(0.35–1.04)	0.31 **	(0.15–0.63)
Self-reported health	0.96	(0.54–1.71)	1.23	(0.64–2.35)	2.98	(0.96–9.21)
**Clinical variables**						
Social participation scores at baseline	1.27 ***	(1.21–1.34)	1.48 ***	(1.40–1.56)	1.98 ***	(0.18–2.14)
Mental health	1.01	(0.96–1.07)	1.05	(0.99–1.11)	1.09 *	(1.00–1.18)
ADL score	1.02	(0.81–1.30)	0.97	(0.67–1.40)	0.73	(0.38–1.39)
IADL score	1.07 *	(1.00–1.04)	1.11 *	(1.02–1.20)	1.03	(0.84–1.26)
CMMSE score	1.05	(0.00–1.11)	1.08 *	(1.00–1.16)	1.17 *	(1.00–1.35)

^1^: The Chinese nation is a big family made up of 56 ethnic groups, most of whom belong to the Han Chinese, while a small number belong to minorities. ADL: Activities of daily living; IADL: Instrumental activities of daily living; CMMSE: Chinese version of modified Mini-Mental State Examination. * *p* < 0.05; ** *p* < 0.01; *** *p* < 0.001.

## Data Availability

Publicly available datasets were analyzed in this study. This data can be found at: https://opendata.pku.edu.cn/ (accessed on 25 September 2022).

## Supplementary Materials

The following supporting information can be downloaded at: https://www.mdpi.com/article/10.3390/ijerph20054260/s1, Figure S1: Trajectories in social participation—Sensitivity analyses (n = 704); Table S1: Baseline characteristics of the total sample between extracted and included participants.
