# Supplementary material for: Trajectories of Social Participation and Its Predictors in Older Adults: Based on the CLHLS Cohorts from 2002 to 2018
Source: Int J Environ Res Public Health. 2023 Feb 27;20(5):4260. doi: 10.3390/ijerph20054260 (PMC10001875; doi:10.3390/ijerph20054260)
Supplement: Supplementary file 1 [file ijerph-20-04260-s001.zip › ijerph-2175139-supplementary.pdf]

Trajectories of social participation and its predictors in older adults: based on the CLHLS cohorts from 2002 to 2018

Table S1. Baseline characteristics of the total sample between extracted and included participants.

Variable	Participants		*p**
	Extracted (n = 13572)	Included (n = 2492)	
**Socio-demographic variables**	88.46 (11.02)	74.69 (7.80)	
Age, Mean(SD)			0.000
Gender			0.000
male	5671 (41.8)	1174 (47.1)	
female	7901 (58.2)	1318 (52.9)	
Ethnic group[1]			0.000
Han Chinese	12860 (94.8)	2304 (92.5)	
Minorities	707 (5.2)	188 (7.5)	
Current marital status			0.000
currently married	3390 (25.0)	1352 (54.2)	
separated	227 (1.7)	69 (2.8)	
divorced	75 (0.6)	19 (0.8)	
widowed	9698 (71.5)	1033 (41.5)	
never married	182 (1.3)	19 (0.8)	
Years of schooling, Mean(SD)	2.62 (8.74)	1.46 (0.50)	0.000
Residence			0.000
city	3426 (25.2)	419 (16.8)	
town	3017 (22.2)	532 (21.3)	
rural	7129 (52.5)	1541 (61.8)	
Co-residence of interviewee			0.000
with household member(s)	11017 (81.2)	2146 (81.6)	
alone	1856 (13.7)	305 (12.2)	
in an institution	699 (5.2)	41 (1.6)	
Pension			0.000
yes	2688 (19.8)	543 (21.8)	
no	10873 (80.1)	1947 (78.1)	
**Independent variables**			
Self-reported quality of life			0.000
high	11389 (83.9)	2315 (92.1)	
low	889 (6.6)	152 (6.1)	
Self-reported health			0.000
healthy	10057 (74.1)	2209 (88.6)	
unhealthy	2228 (16.4)	259 (10.4)	
Currently smoking			0.000
no	11222 (82.7)	626 (25.1)	
yes	2319 (17.1)	1866 (74.9)	
Currently drinking			0.000

no	10871 (80.1)	595 (23.9)	
yes	2668 (19.7)	1896 (76.1)	
Hearing, Mean(SD)	2.26 (1.09)	1.12 (0.47)	0.000
Visual function, Mean(SD)	2.43 (0.96)	1.18 (0.56)	0.000
Fruit and vegetables intake, Mean(SD)	3.48 (1.44)	3.70 (1.21)	0.000
Mental health, Mean(SD)	23.33 (12.11)	19.89 (3.91)	0.000
ADL, Mean(SD)	10.63 (2.57)	11.89 (0.64)	0.000
IADL, Mean(SD)	9.02 (6.09)	14.58 (2.95)	0.000
CMMSE, Mean(SD)	15.69 (7.42)	20.60 (3.48)	0.000
**Dependent variables**			
Baseline social participation, Mean(SD)	13.08 (6.40)	13.95 (5.58)	0.000

[1]: The Chinese nation is a big family made up of 56 ethnic groups, most of whom belong to the Han Chinese, while a small number belong to minorities. ADL: Activities of daily living; IADL: Instrumental activities of daily living; CMMSE: Chinese version of modified Mini-Mental State Examination. *Student's t-test. The sum of percentages may not equal 100% due to the availability of missing data.

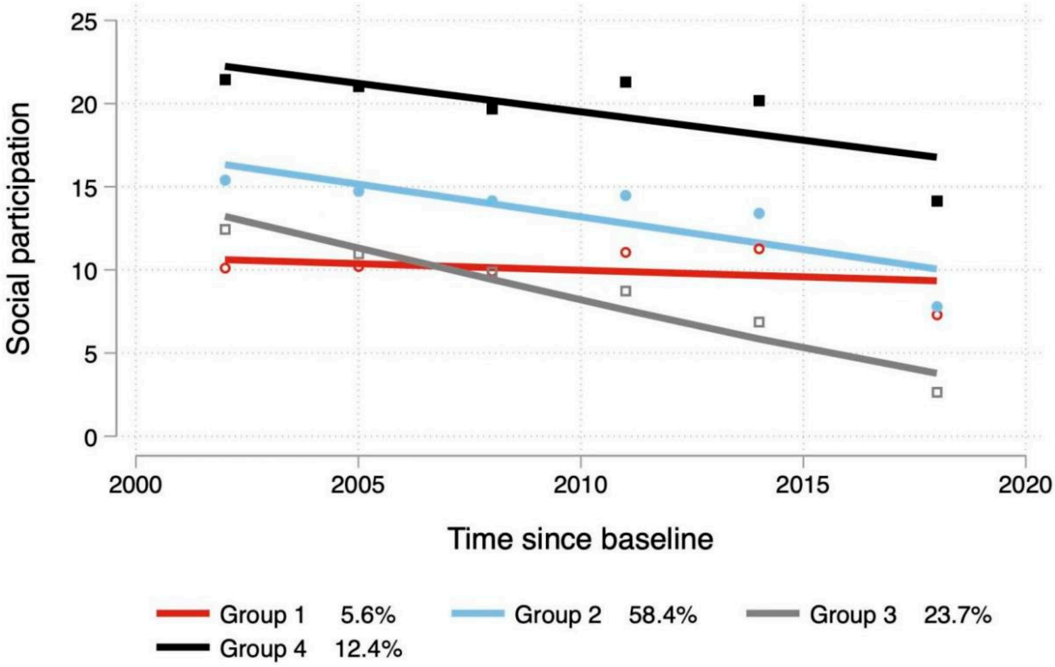

Figure S1. Trajectories in social participation - Sensitivity analyses (n = 704)
